# Molecular characterization of the type species of *Kyrtuthrix* (Rivulariaceae, Cyanobacteriota) with comparison to *Nunduva*: Morphologically different but molecularly cryptic genera

**DOI:** 10.1111/jpy.70063

**Published:** 2025-07-28

**Authors:** Alžběta Vondrášková, Tomáš Hauer, Jan Mareš, Esther Berrendero‐Gomez, Jan Zima, Haydee Montoya‐Terreros, Chelsea D. Villanueva, Jeffrey R. Johansen

**Affiliations:** ^1^ Faculty of Science University of South Bohemia České Budějovice Czech Republic; ^2^ Institute of Hydrobiology Biology Centre of the Czech Academy of Sciences České Budějovice Czech Republic; ^3^ Institute for Agro‐Food and Agro‐Environmental Research and Innovation, Miguel Hernández University of Elche (CIAGRO‐UMH) Elche Spain; ^4^ Museo de Historia Natural UNMSM Jesús María, Lima Peru; ^5^ Department of Biology John Carroll University University Heights Ohio USA

**Keywords:** 16S rRNA, 16S‐23S ITS, intertidal zone, multilocus analyses, *rbc*LX, *rpo*C1, supratidal zone, type locality, type species

## Abstract

The euendolithic genus *Kyrtuthrix* was originally described in 1929 by A. Ercegović from the Dalmatian coast. Due to its isopolar filaments, *Kyrtuthrix* was classified within the cyanobacterial system as part of the family Scytonemataceae, even though trichomes tapering toward their ends represent a feature typical of the family Rivulariaceae. In the last decade, four new species of *Kyrtuthrix* have been described. Their sequences helped to establish *Kyrtuthrix* as belonging to the family Rivulariaceae. However, the new species demonstrated that *Kyrtuthrix* was not always euendolithic, as the new discoveries occurred on igneous rocks that were not susceptible to penetration by cyanobacteria. We were able to obtain sequences of the 16S rRNA gene, the 16S‐23S ITS rRNA region, the *rpo*C1 gene, and *rbc*LX gene for phylogenetic analyses of two classical species of *Kyrtuthrix—*the type species *K. dalmatica* collected from the Dalmatian coast and *K. maculans* collected from the Pacific coast of Peru. Our analyses revealed that both taxa were related to the newly described *Kyrtuthrix* species, although they were not clearly separated by molecular character sets from the more recently described *Nunduva*. A third *Kyrtuthrix* species from the coast of France was characterized by us during this study but was intermediate in morphological structure and phylogeny between *K. dalmatica* and *K. maculans* and not given a specific epithet. If we relied on the 16S rRNA gene sequence evidence, *Nunduva* would have been collapsed into the older genus *Kyrtuthrix.* However, using multiple gene evidence, they formed sister clades and, therefore, have been treated as distinct genera in this manuscript.

**Abbreviations**
ASM: artificial seawater medium
BI: Bayesian Inference
DIV: D1 index value
ESS: estimated sample size
GTR + I + G: general time reversible model with a proportion of invariable sites and gamma distribution
ML: maximum likelihood
MP: maximum parsimony
OTU: operational taxonomic unit
PCR: polymerase chain reaction
PSRF: potential scale reduction factor

## INTRODUCTION

In the last 2 decades, a large number of sequences of various genes and whole genomes of cyanobacteria have been produced. Unfortunately, in many cases, the source organisms have not been determined correctly, or the sequences of environmental material have been named according to the most similar sequence retrieved using a BLAST search in the main sequence repositories (DDBJ/EMBL/GenBank). These incorrect name assignments consequently lead to subsequent incorrect identifications, triggering a chain reaction of mistakes. This phenomenon has implications for the study of evolutionary relationships and the subsequent construction of the taxonomic system of the phylum. In order to make correct generic assignments, whether that is to existing classical genera or to genera new to science, taxonomists need sequence information for the type species of classically described genera. This is often difficult because most of these generitypes are not currently in culture, and special efforts must be made to either find and isolate the types so that they can be sequenced or to find and sequence the types directly from environmental samples. Successful efforts in this vein have been made for the types of some important genera: *Oscillatoria princeps* (Mühlsteinová et al., 2018), *Stigonema mamillosum* (Vondrášková et al., 2024), *Capsosira brebissonii* (Vondrášková et al., 2024), *Pleurocapsa fuliginosa* (Shalygin et al., 2019), *Nostoc commune* (Řeháková et al., 2007), *Umezakia natans* (McGregor et al., 2023), and *Mastigoladus laminosus* (Kaštovský & Johansen, 2008), to name a few examples. However, some genera appear polyphyletic in phylogenetic trees, and the revision of these genera is dependent upon finding and sequencing the type species. An excellent example of this problem is our inability to find and sequence the type species of *Calothrix, C. confervicola*, leaving many strains to be assigned to this genus based on the solitary tapering trichomes (Kumar et al., 2022; Saraf et al., 2024). To obtain a robust and stable taxonomy, the information on nomenclatoric types should be as broad as possible in terms of molecular data and should include detailed morphology of cultures and natural material. Last but not least, the ecology of the material designated as a reference must fit the original description.

There are two validly classically described species in *Kyrtuthrix*: *K. dalmatica*, the generitype described by Ercegović  (1929) from Croatia, and *K. maculans*, from Thailand under the name *Brachytrichia maculans* in West et al. (1901) and later combined into *Kyrtuthrix* by Umezaki (1958). The three species described in the last decade were all from Mexican coastal sites: *K. huatulcensis* by León‐Tejera et al. (2016) and *K. munecosensis* and *K. totonaca* by Johansen et al. (2021). Members of the genus have been present in tropical to temperate marine coastal waters all over the world. They have been reported from intertidal or supralittoral zones of seas and oceans, in Peru (Montoya Terreros, 2003), Japan (Umezaki, 1958), Croatia (Brandes et al., 2015; Vondrášková et al., 2017), and South Africa (Silva & Pienaar, 2000), for example. Morphology, especially the form of branching, and the endolithic activity has been studied by several authors (Golubic & LeCampion‐Alsumard, 1973; Umezaki, 1958).

Here we have contributed to cyanobacterial systematics with the molecular characterization of the type species *Kyrtuthrix dalmatica*, using sequences obtained from material collected in the *locus classicus* mentioned by Ercegović  (1929). Additionally, we have provided a comparison to other members of the genus: *K. maculans*, reference sequences of recently described *Kyrtuthrix* species mentioned above, and the recently described genus *Nunduva* (González‐Resendiz et al., 2018) as the nearest related taxon, possibly congeneric with *Kyrtuthrix*.

## MATERIALS AND METHODS

### Sampling and cultivation

Strain Cr5 of *Kyrtuthrix dalmatica* was isolated from a sample collected in September 2014, from the type locality, Čiovo Island, Croatia, 43°29′39.7″ N, 16°14′44.503″ W. An unnamed *Kyrtuthrix* species (strain N3) was isolated from a sample from a tidal pool on the Atlantic coast in France, near Goury, 49°43′20.176″ N, 1°56′44.070″ W, and a strain matching *K. maculans* (strain PE16) was collected from Playa La Mina, Paracas, Peru, 13°54′41.060″ S, 76°19′4.793″ W. Fragments of the rock, several cm in size, were taken using a geological hammer at the lower part of the supratidal, allowed to dry, and marked. Before microscopy, the dried samples were refreshed in sterile seawater (from the sampling locality) for about a week in a cultivation room where the temperature was maintained at 21°C, and light conditions were on a 12:12 h light:dark cycle with a light intensity 16 μmol photons · m^−2^ · s^−1^. To prepare a mount without any calcite particles, the samples were dissolved by placing stone samples in 0.1 M HCl. Mounts were then inspected using an Olympus BX 51 photomicroscope with a digital camera Olympus DP‐71. Pictures were taken with the software DP Controller 3.1 (Olympus Corp., Tokyo, Japan) and QuickPHOTO MICRO 3.0 (Promicra, Prague, Czech Republic). Parts of the samples were placed in Petri plates with artificial seawater medium (ASM; Sera Marine Salt, Heinsberg, Germany) solidified with 1.5% agar. Crude cultures were checked every 3 weeks, and pure filaments or colonies were transferred repeatedly to new plates so long as the biomass was unialgal. The strains were transferred to tubes containing solid ASM and watered with a few milliliters of ASM. Tubes with cultures were kept in the same conditions as were in the cultivation room as described above.

### Molecular analyses

DNA for the following analyses was extracted from dried biomass with the NucleoSpin Plant II, Mini kit for DNA from plants (Machery‐Nagel, Düren, Germany). The genomic DNA served as a template for amplifying three genes using standard primers and protocols; for the 16S rRNA gene and the associated 16S–23S ITS rRNA region, we followed Mühlsteinová et al. (2018); for the *rbc*LX gene, Rudi et al. (1998); and for the *rpo*C1 gene, Seo and Yokota (2003). All polymerase chain reaction (PCR) products were subsequently cloned following Mareš et al. (2015). Plasmids with an insert were commercially sequenced by SEQme (https://www.seqme.eu/en/), using the Sanger dideoxy sequencing method. The sequences were submitted to the NCBI GenBank database (https://www.ncbi.nlm.nih.gov/genbank/about/).

Several different phylogenetic analyses were conducted. First, an alignment with the in‐group (Rivulariaceae sequences) with several Nostocales plus two *Chroococcidiopsis* sequences as outgroup taxa was constructed with sequences having a minimum length of 1161 nucleotides and a maximum sequence length of 1412 nucleotides using ClustalW (https://www.genome.jp/tools‐bin/clustalw, accessed December 1, 2024) with manual correction supported by secondary structure. Bayesian inference (BI) analysis was done using the GTR + I + G evolutionary model, a model repeatedly shown to be the best model for cyanobacterial ribosomal sequences, and was performed using MrBayes 3.2.7a (Huelsenbeck & Ronquist, 2001) on the CIPRES Science Gateway with 95 million generations, sampling every 1000 generations, utilizing BEAGLE (Ayres et al., 2012), and achieving an average standard deviation of split frequencies = 0.035 with an estimated sample size (ESS) > 100 for all parameters and a potential scale reduction factor (PSRF; Gelman & Rubin, 1992) approaching 1.00 for all parameters. This phylogeny established what sequences belonged to Rivulariaceae and Calotrichaceae (tree not shown). A second analysis was performed with just the in‐group (57 Rivulariaceae) sequences. This analysis achieved an average standard deviation of split frequencies <0.010, with ESS > 800 and PSRF = 1.000 for all parameters. A maximum‐likelihood (ML) analysis using the same evolutionary model was performed in RaxML 8.2.12 (Stamatakis, 2014) and also on the CIPRES Science Gateway. Bootstrap values from this analysis were mapped onto nodes of the BI analysis.

An alignment of 18 ITS rRNA region sequences of *Kyrtuthrix* and *Nunduva* species was constructed, and two analyses were run on this alignment. First, a BI analysis was run with two partitions, one for DNA sequence data and one for standard data, which consisted of coding for indels in the alignment (0 = missing nucleotide, 1 = nucleotide present). This tree had an average standard deviation of split frequencies <0.001 and PSRF = 1.000 for all parameters. Second, a maximum parsimony analysis was run in PAUP 4.a168 (Swofford, 2002) with indels coded as a fifth base. Bootstrap values from this analysis were mapped to the nodes of the BI analysis. Using this alignment, we also determined percent dissimilarity among ITS rRNA regions by determining p‐distance in PAUP and then applying the formula 100 × p.

Analysis of the 16S rRNA, *rbc*LX, and *rpo*C1 genes in a concatenated 2502‐bp long alignment consisting of 73 operational taxonomic units (OTUs) was performed (both BI and ML, as above) for those taxa containing all three genes. This tree separated *Kyrtuthrix* from *Nunduv*a but did not include all species, as most species only had 16S rRNA gene data (Figure S1). We then ran another ML 16S rRNA gene analysis, but it was constrained by the three‐gene tree results, that is, we had to force *K. huatulcensis* to be sister to *K. dalmatica* and *K. maculans*. This analysis was based on an alignment of 197 taxa. We conducted a BI analysis with the same alignment and mapped the posterior probabilities of this analysis onto the constrained ML tree (Figure S2).

All trees were visualized in FigTree v1.4.4. Trees were then post‐edited in Adobe Design Standard CS5. The p‐distance based on the 16S rRNA gene sequence was calculated for the 1412 nt alignment in PAUP on ACCESS (4.a168) and reported as percent similarity [100 × (1 − p)]. Conserved helices of the 16S–23S ITS rRNA region were determined for the *Kyrtuthrix* and *Nunduva* strains for which ITS rRNA region sequence data existed in NCBI. Structures were estimated in Mfold, located on the UNAfold website (unafold.org, Zuker, 2003), and redrawn in Adobe Illustrator. To test for the presence of paralogous operon copies, D1 index values (DIV) were calculated following the methodology outlined in Villanueva et al. (2024). Nucleotide pairs forming the backbone of helices were categorized and summed. Non‐canonical base pairs at specific helical loci were compared among nearest neighbors per the BI ITS rRNA region tree to classify transitions as alpha or beta (see Figure S3 for details). D1 index values were plotted against total helix length (nt; Figure S4).

## RESULTS

### Morphological characterization

**FIGURE 1:**
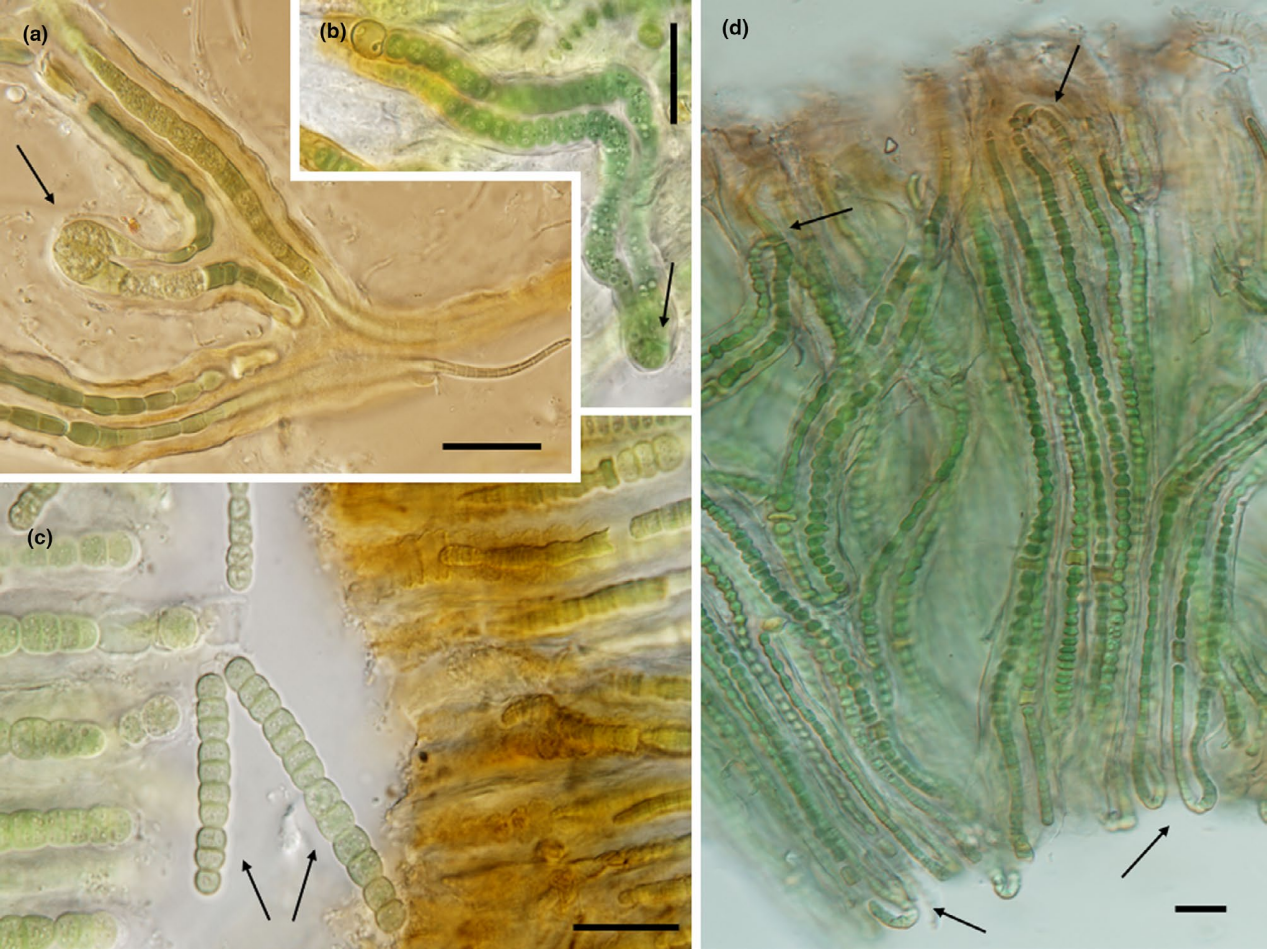
Morphology of *Kyrtuthrix* species. (a) *K. dalmatica* Cr5 filament with lateral loop (arrow) from nature. (b) *Kyrtuthrix* sp. N3 U‐form filament (arrow) from culture. (c) *K. maculans* PE16 hormogonia (arrows) from nature. (d) *K. dalmatica* Cr5 typical parallel organization of U‐form filaments (arrows) in colony with yellow‐brown sheet ends (from nature). Scale = 20 μm.

Three strains were obtained in the course of this study: *Kyrtuthrix dalmatica* Cr5 from Croatia, *Kyrtuthrix maculans* PE16 from Peru, and *Kyrtuthrix* sp. N3 from France (Figure 1). The type species of *Kyrtuthrix*, *K. dalmatica*, formed an epilithic colonial cushion of filaments embedded in mucilage, with some filaments penetrating the calcareous substrate. Filament length was 300–350 μm. Trichomes grew in dense, parallel fascicles, more or less perpendicular to the substrate, regularly forming loops in the basal attached portion of the colony, but also with loops in the surface portion of the colony as well as lateral loops in the central region of the colony (Figure 1a,d). Trichomes tapered toward both ends (isopolar) but could appear to be tapered at only one end (heteropolar) when fragmenting. Trichomes were bright blue‐green in colorless mucilage, although mucilage became brownish at the surface, with clear constrictions at the cross walls. Cells were bead‐like and isodiametric throughout most of the trichome, but could become elongated and irregular in form (Figure 1a), when actively growing within the mucilage 6.0–8.4 μm wide, 4.8–6.0 μm long, when tapered at the apices 1.0–2.0 μm wide, 2.0–3.0 μm long. Heterocytes were infrequent, intercalary, solitary, barrel shaped, 4.8–8.0 μm wide, 4.8–11.9 μm long, or oval, 7.0–8.5 μm in diameter. Hormogonia were not observed. This morphology was fully consistent with the original description of Ercegović  (1929) and the strain was collected from the type locality. We have high confidence that our topotype strain is within the *K. dalmatica* lineage. Herbarium voucher deposited as CBFS A258‐1.

*Kyrtuthix maculans* PE16 had a blackish, wide spreading colony, with trichomes embedded in colorless mucilage, which became brownish in the surface region (Figure 1c). Trichomes were arranged in dense parallel fascicles 130–200 μm long, forming loops in the basal portion as well as in the surface layer, clearly constricted at the cross walls, tapered toward the apices, producing hormogonia in 22–58 μm length. Cells were blue‐green to olive in color, mostly isodiametric, but both shorter and longer than wide, 3–6 μm wide, (4)–7–12 μm long (3–4 μm wide, 4–5 μm long when tapering). Heterocytes were nearly spherical, 9 μm in diameter, or barrel shaped, 6.5–9 μm wide, 6–12 μm long. Hormogonia when observed were 22–58 μm long. This strain fit the description of *K. maculans*, although there were practically no diagnostic morphological features that separate this species from *K. dalmatica*. Its distribution in the Pacific Ocean along the coastline of Peru was somewhat closer to the type locality for *K. maculans* in Southeast Asia than to the Mediterranean where *K. dalmatica* was described. For the rest of this paper, we will refer to this strain as representative of *K. maculans*. Herbarium voucher deposited as CBFS A259‐1.

The third strain, *Kyrtuthrix* sp. N3, had very similar morphology to both *K. dalmatica* and *K. maculans* (Figure 1b). Filaments were 175–250 μm long. Cell width measured 5–8 μm, with the length 4–10 μm (but was 3–4 μm wide, 3–5 μm long when tapering). Heterocytes were oval, 6–9 μm in diameter, barrel‐shaped 5–12 μm wide, 5–10 μm long. Since it is from the Atlantic coast of France, it is ecologically separated from *K. dalmatica*, which occurs in the more saline waters of the Mediterranean Sea, and it is ecologically separated from the tropical and subtropical waters of the other four species described to date.

### Molecular characterization

For *Kyrtuthrix dalmatica* Cr5 (Figure 1a,d), type species from type locality, we obtained seven cloned sequences of the 16S rRNA gene. For the molecular analyses, only one sequence was used—the reference sequence PQ627892, which was identical to the consensus sequence constructed from all cloned sequences. All the sequences are available on GenBank under the following accession numbers: 16S rRNA PQ627887–PQ627893; 16S‐23S ITS PQ627894; *rbc*LX PQ625366–PQ625368; and *rpo*C1 PQ625362. For *Kyrtuthrix maculans* PE16 (Figure 1c), we obtained seven cloned sequences of the 16S rRNA +16S–23S ITS rRNA region. For the molecular analyses, only one sequence was used—the reference sequence PQ627901, also identical to the consensus sequence constructed from all cloned sequences. Sequences are available on GenBank as follows 16S rRNA+16S‐23S ITS PQ627895–PQ627901; *rbc*LX PQ625370; and *rpo*C1 PQ625365. For *Kyrtuthrix* sp. N3 (Figure 1b), only seven sequences of the 16S rRNA gene were obtained: PQ608605–PQ608611. PQ608610 was identical to the consensus sequence constructed from all cloned sequences and hence was chosen as the reference sequence. Other genes were not successfully amplified as the culture did not remain in a viable condition for long. Additionally, we amplified additional sequences from *Kyrtuthrix huatulcensis* C708 (*rpo*C1 PQ625364) and *Nunduva britannica* (*rpo*C1 PQ625363, *rbc*LX PQ625369).

**FIGURE 2:**
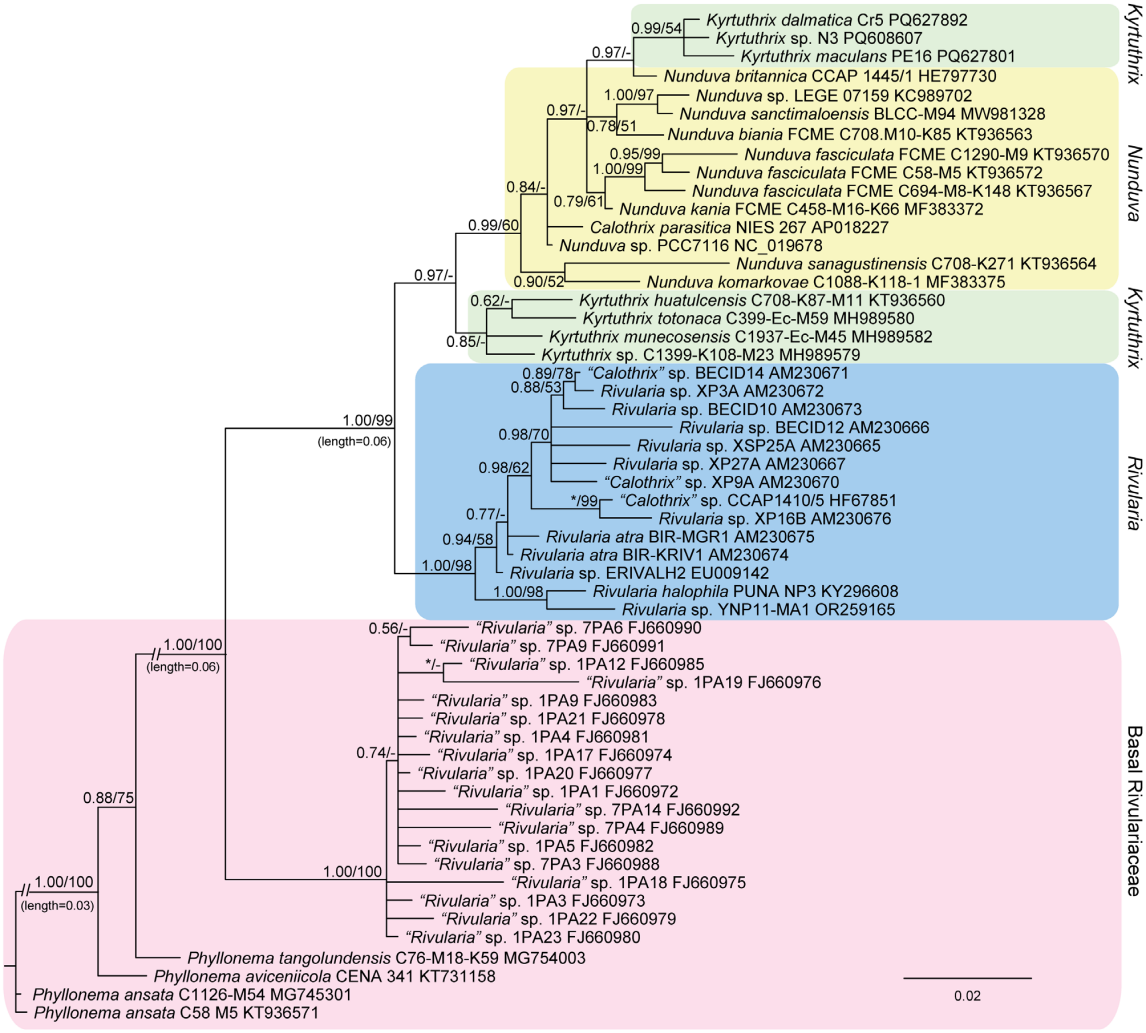
BI phylogeny based on the 16S rRNA gene sequence of 57 strains within the Rivulariaceae according to generic placement. Shaded boxes are groups labeled to the right. The Basal Rivulariaceae consist of *Phyllonema* species and a group of misidentified *Rivularia* strains. Note that *Kyrtuthrix* is not monophyletic in this analysis. Posterior probabilities from the BI analysis and bootstrap values ≥50% from the ML analysis are mapped to nodes.

**FIGURE 3:**
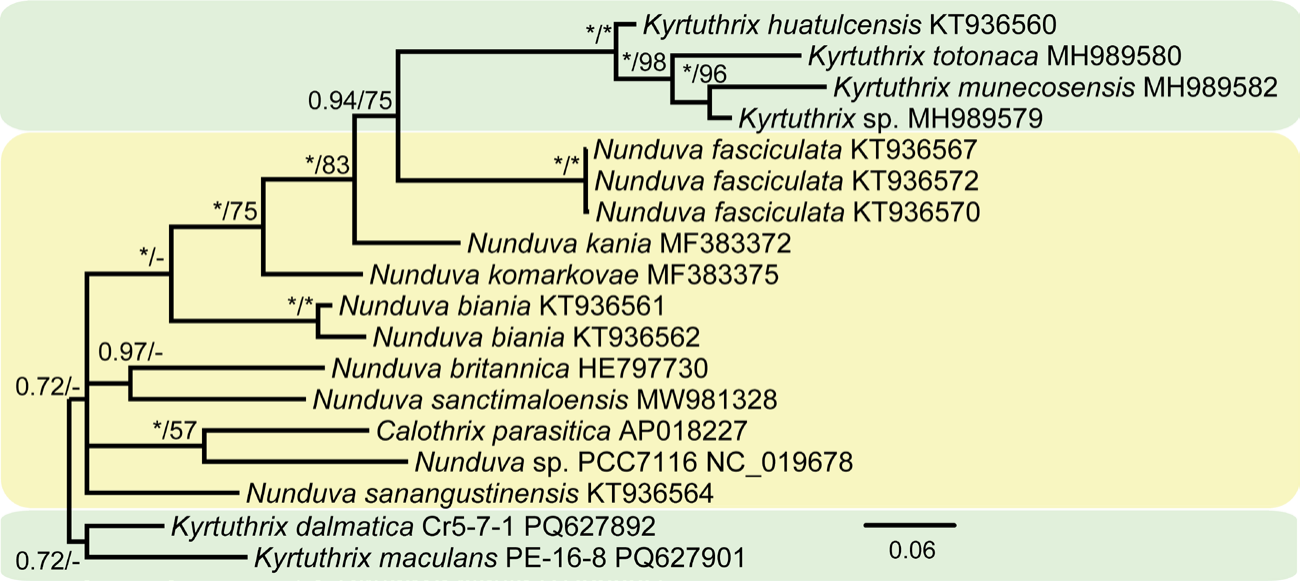
BI Phylogeny based on aligned 16S‐23S ITS rRNA regions. *Kyrtuthrix* is again separated into two clades, with *Nunduva* nested within *Kyrtuthrix*. BI posterior probabilities (* = 1.00) and MP bootstrap values ≥50% are mapped to nodes (* = 100%).

The 16S rRNA gene phylogeny based on 197 Nostocales showed the Rivulariaceae as a well‐supported node in the Nostocales. All 57 Rivulariaceae identified in the analysis were then analyzed to produce a second 16S rRNA gene phylogeny (Figure 2). The topography of the Rivulariaceae was identical in the two analyses. All *Kyrtuthix* and *Nunduva* species were clearly in the family Rivulariaceae, as has been established in previous papers (González‐Resendiz et al., 2018; Johansen et al., 2021; León‐Tejera et al., 2016). However, the 16S rRNA phylogeny did not separate *Nunduva* from *Kyrtuthrix* (Figure 2). The three species of *Kyrtuthrix* sequenced in this effort were phylogenetically displaced from the other three *Kyrtuthrix* species isolated from Mexican coasts, with all *Nunduva* species falling between the two *Kyrtuthrix* clusters. If a taxonomic decision on the status of these two genera were based only on 16S rRNA gene phylogeny, then all *Nunduva* species would be transferred to *Kyrtuthrix* via new combinations, as *Kyrtuthrix* is the taxon with priority. Percent similarity (PS) of 16S rRNA gene sequences also supported collapsing the two genera, with PS ≥ 97.0% for all two‐way comparisons (Table S1). Most of the species were not resolved well, based on the 16S rRNA gene threshold for species delineation (≤98.7%). However, percent dissimilarity (PD) of the 16S–23S ITS rRNA region fully supported separate species in both genera (Table S2). The phylogenetic analysis of the aligned ITS rRNA region sequences was in agreement with the 16S rRNA gene BI analysis in separating *K. dalmatica* and *K. maculans* from the Mexican coast *Kyrtuthrix* species (Figure 3).

**FIGURE 4:**
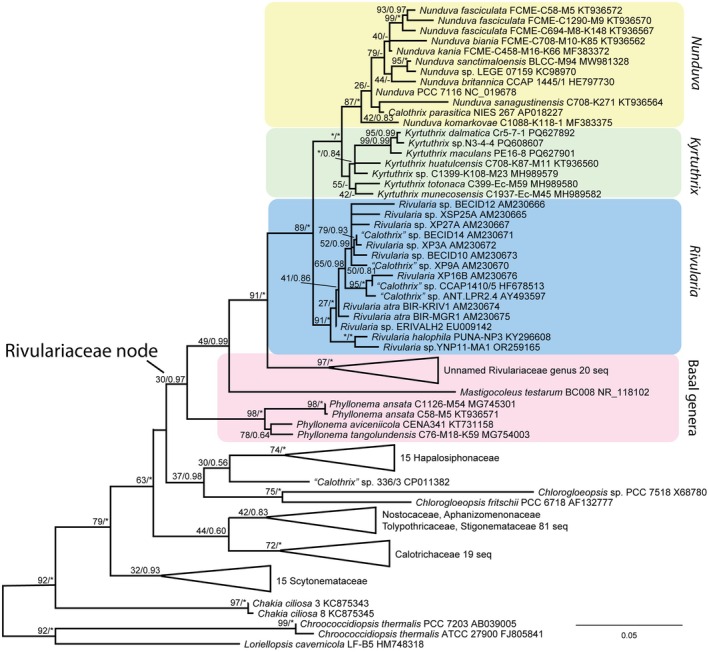
ML phylogeny based on 16S rRNA gene sequence constrained by the phylogeny based on a concatenated alignment of three genes (16S rRNA, *rbc*LX, and *rpo*C1). *Kyrtuthrix dalmatica* and *K. maculans* were forced to be sister to *K. huatulcensis* to achieve the constraint; all other sequences were not constrained. BI posterior probabilities are mapped to the nodes; * = 100% for ML bootstrap support and * = 1.00 for posterior probability support.

In the reduced‐taxon three‐gene analysis (Figure S1), *Kyrtuthrix* and *Nunduva* were clearly separated into supported sister clades in the family Rivulariaceae, with *Phyllonema* still in a basal position to *Kyrtuthrix* and *Nunduva*. Their severance was 100% supported in both BI and ML analyses. *Kyrtuthrix huatulcensis* was joined to *K. dalmatica* and *K. maculans*. When we reran the 16S rRNA gene ML analysis with the topology of that tree constrained by the three‐gene tree, all *Kyrtuthrix* species formed one node, and all *Nunduva* species formed a sister node (Figure 4 and Figure S2). This analysis supported keeping the two genera separate.

### Secondary structure of the ITS rRNA region

**FIGURE 5:**
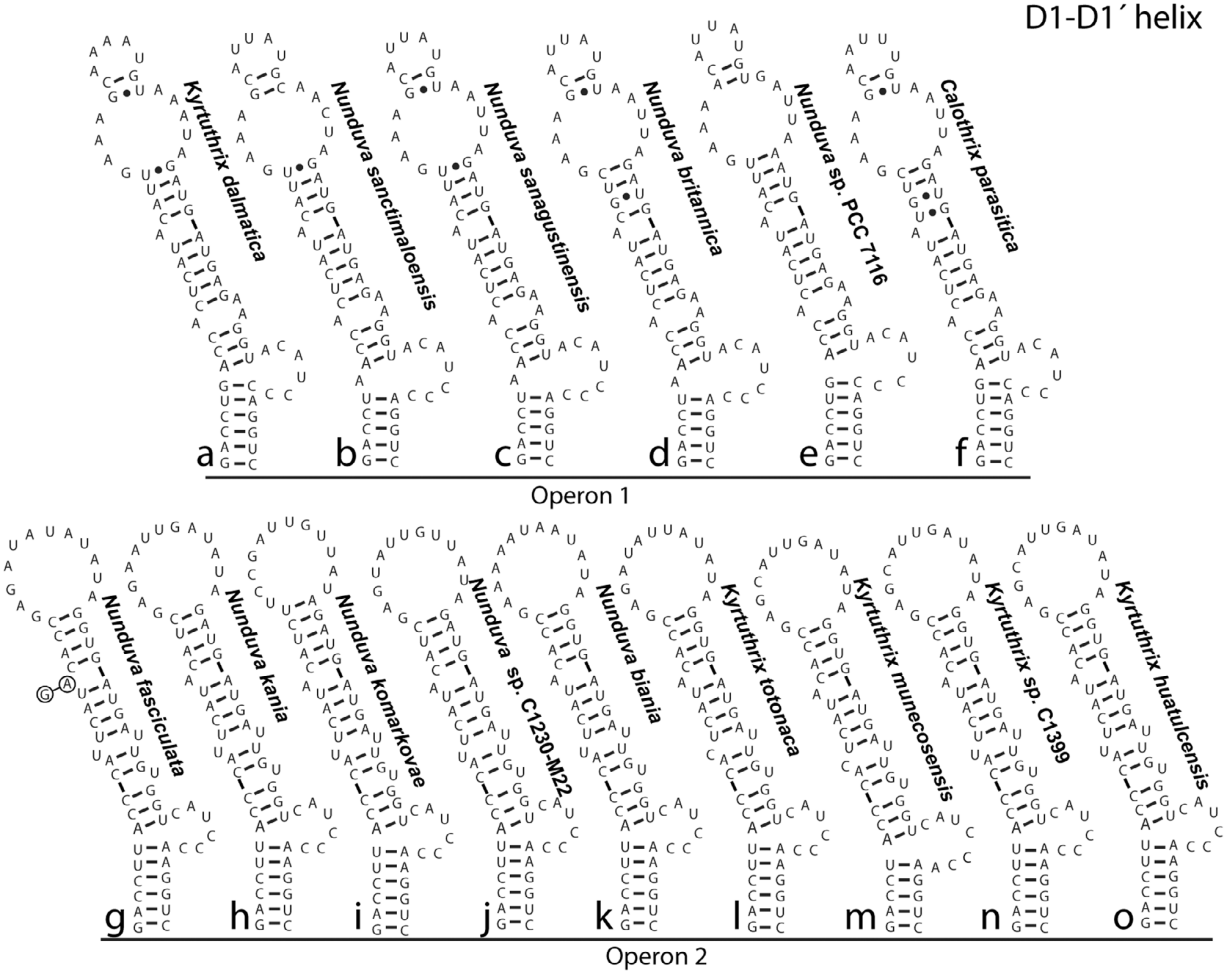
Secondary structure of the D1–D1′ helix, arranged by operon type, with species designations to the right of each figure. *Nunduva fasciculata* had two different sequences for this domain (differing nucleotides circled), but they had identical structure.

All 16S‐23S ITS rRNA regions observed in this study had both tRNA genes, tRNA^Ile^ and tRNA^Ala^. The D1–D1′ helices had two different motifs among the different species (Figure 5; Table S3). *Kyrtuthrix dalmatica, Nunduva sanctimaloensis, N. sanagustinensis, N. britannica*, *Nunduva* sp. PCC 7116, and *Calothrix parasitica* NIES 267 all had D1–D1′ helices 64 nucleotides long, with a small (5 nu) terminal loop separated from a large bilateral bulge by two base pairs, and a bilateral bulge near the basal end consisting of a mismatch of three adenine residues (Figure 5a–f). These structures were designated as operon 1. The four Mexican coast *Kyrtuthrix* species, *K. totonaca, K. munecosensis, K. huatulcensis*, and an unspeciated *Kyrtuthrix*, along with all remaining *Nunduva* species, had D1–D1′ helices 60–62 nucleotides in length, with a large (12–13 nucleotides) terminal loop without a subterminal bilateral bulge, and two mismatches near the basal end, including an unmatched uracil upstream from a uracil/uracil mismatch (Figure 5g–o). These structures were designated operon 2. Both operons shared high sequence similarity with the notable exception that helices of operon 1 included non‐canonical base pairs (G•U/U•G indicated by black circles instead of dashes), whereas helices of operon 2 had none. This dichotomy in structures suggested the presence of paralogous operons, but in no case did we find both of these structures in a single species or strain. In *Calothrix parasitica* there were two different operons, but both belonged to operon type 1. Multiple operons were not observed in any other strain, but we suspect this may occur. The D1–D1′ helix was incomplete in *K. maculans*, but the portion sequenced suggested it possesses operon type 1. Only when multiple clones from multiple strains are sequenced does the opportunity exist to identify these operons, yet studies done elsewhere have shown that within genera, the operon number is consistent within species.

To test for the presence of paralogous operon copies, we determined DIVs for D1–D1′ helices from operons 1 and 2. The DIVs calculated for helices of operon 1 were more variable, ranging from 2.09 to 2.93, than those of operon 2, which ranged from 2.69 to 2.94. These values were midrange in comparison to values recovered for *Brasilonema* operons (Villanueva et al., 2024, see table 3). When DIVs were plotted against total helix length, there was some degree of separation between operons 1 and 2 (Figure S4). However, DIVs for both operons were very similar, and separation among operons was mostly due to variations in helix length. Although the operons may be diverging, they have not diverged enough to consider them paralogous.

**FIGURE 6:**
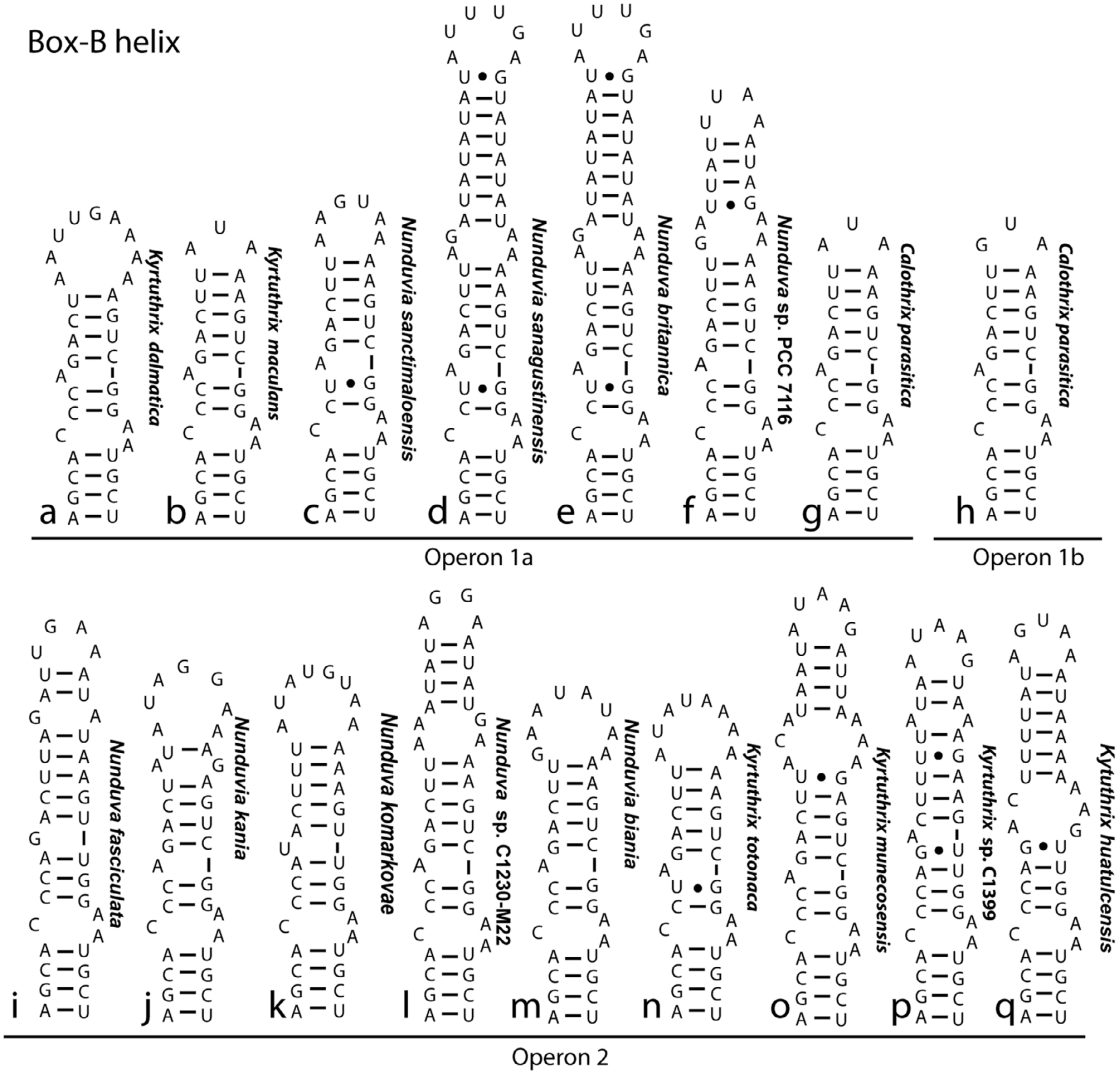
Secondary structure of the Box‐B helix, arranged by operon type, with species designations to the right of each figure. *Calothrix parasitica* had two different operons, with type 1b being very similar to type 1a.

The Box‐B helices were variable in length, structure, and sequence and did not sort into the same groups as the D1–D1′ helices (Figure 6). *Kyrtuthrix dalmatica* had a shortened helix similar to *K. totonaca, K. maculans, Nunduva kania, N. komarkovae, N. biania*, and *N sanctimaloensis* (Figure 6a–c,g,h,j,k,m,n). The other species had similar sequences and structures to the *K. dalmatica* group but were extended into longer helices by insertion of additional uracil and adenine nucleotides (Figure 6d–f,i,l,o–q). The ancestral Box‐B likely was a shorter helix, which was extended in several lineages to produce the longer helices.

**FIGURE 7:**
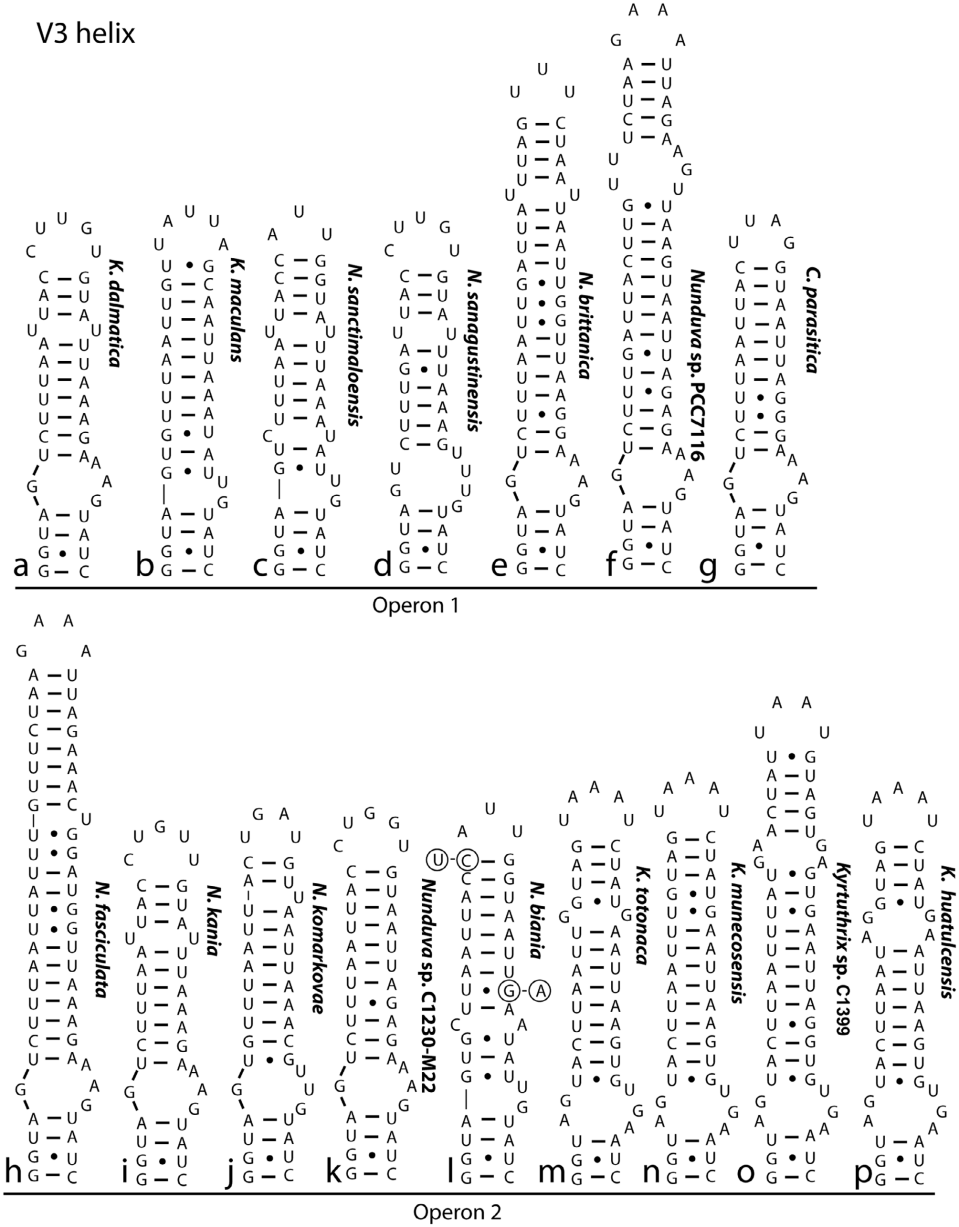
Secondary structure of the V3 helix, arranged by operon type, with species designations to the right of each figure. The strains of *Nunduva biania* showed some sequence variability among strains, although the structure was identical. Non‐canonical base pairings (U•G/G•U) indicated by circles instead of dashes.

The V3 helices had a uniform four base pair basal clamp, 5′‐GGUA–UAUC‐3′, which was shortened to a three base pair clamp in several of the *Kyrtuthrix* species (Figure 7). The helix between the basal bilateral bulge and the terminal loop also had many similarities between species. The most similar pair of helices was those for *K. dalmatica* and *Nunduva kania*, which differed only by a single nucleotide in the terminal loop. There were multiple nucleotide differences in all other V3 helices, including significant differences in length.

### Designation of a nomenclatural type

*Kyrtuthrix dalmatica* Ercegović  1929.

The specimens we analyzed were collected at the type locality at Čiovo Island, Croatia. The population we sampled fit the description of this genus and species well (Ercegović, 1929; Komárek, 2013), and we are confident in the species determination. No holotype was designated by Ercegović, and no original material is known to exist, so it is necessary to designate a lectotype for this species. This lectotype is ambiguous because it is based on a line drawing without original material to examine with microscopy, and furthermore, it lacks molecular sequence data allowing modern taxonomic criteria to be used in its definition.

Lectotype here designated: fig. 3 in Ercegović  (1929). Sur quelques nouveaux types des Cyanophycées lithophytes de la cote adriatique. *Archiv fur Protistenkunde*, 66: 164–174.

Epitype here designated in support of the above lectotype: CBFS A258‐1, air‐dried herbarium preparation containing biomass of the strain Cr5 isolated from Čiovo Island (43°29′39.7″ N, 16°14′44.50.3″ W) in Croatia, coll. A. Vondrášková, September 11, 2015

Reference micrographs based on the epitype material from the Čiovo Island, Croatia (Figure 1a,d).

## DISCUSSION

There is an ongoing debate regarding the taxonomic status of *Kyrtuthrix dalmatica* and *K. maculans*, with some researchers, such as Umezaki (1958), treating them as a single species. The primary difference is that *K. dalmatica* grows endolithically in limestone whereas *K. maculans* is epilithic on impenetrable igneous or metamorphic rock. Our study, which examined both taxa, revealed that although morphological characteristics in culture provided little distinction between them, molecular analyses, particularly of percent dissimilarity in their ITS rRNA region sequences, demonstrated a clear genetic separation. *Kyrtuthrix dalmatica* and *K. maculans* differ in that the former is endolithic whereas the latter is not, at least according to the description in Komárek (2013). Restriction to an endolithic habitat may not be a key determinant in differentiating the two species; rather it may just be related to the available substrate in their unique habitats.

Based on morphology, *Nunduva* is easily distinguished from *Kyrtuthrix*. All species of *Kyrtuthrix* occurred in the intertidal or supratidal zone, with filaments growing in typical U‐form loops, with all filaments in dense, parallel formation in the colony. False branching was not observed in any *Kyrtuthrix* strain. *Nunduva*, conversely, is marine and epilithic, but morphologically different in that its isopolar or heteropolar filaments never form densely arranged, parallel, U‐shaped trichomes in the colony, and it possesses both single and double false branching. The 16S rRNA gene phylogeny supported combining these two genera, in which case all *Nunduva* species would be moved into *Kyrtuthrix*, the genus with nomenclatural priority. The 16S‐23S ITS rRNA region phylogeny also supported combining the two genera, although this phylogeny was constructed with sequences from different operons, invalidating the use of the ITS rRNA region phylogeny to make taxonomic decisions. However, the three‐gene tree supported keeping the two genera separate. Given this conflict in the analyses, we felt it was premature to make a taxonomic transfer. With additional sequencing, particularly of the *rbc*L and *rpo*C1 genes for the Mexican *Kyrtuthrix* species (*K. totonaca* and *K. munecosensis*), this conflict may be resolved. Whole genome sequences would also be very helpful in resolving this taxonomic puzzle.

The Rivulariacean genera *Kyrtuthrix* and *Nunduva* comprise an interesting example in cyanobacteria of genera that are morphologically distinct while phylogenetically cryptic. More often, genera are morphologically cryptic but separated by molecular data, particularly phylogeny and percent similarity of 16S rRNA genes (Alvarenga et al., 2021; Hauerová et al., 2021; Jusko & Johansen, 2024; Mai et al., 2018; Pietrasiak et al., 2019, 2021). Relatively few examples exist of two genera with very different morphologies being shown to belong to the same clade based on 16S rRNA phylogeny; however, two examples are *Raphidiopsis* and *Cylindrospermopsis* (Aguilera et al., 2018) and *Chrysosporum* and *Umezakia* (McGregor et al., 2023). In the case of *Raphidiopsis* and *Cylindrospermopsis*, the loss of heterocytes in the former is tied to the loss of N_2_‐fixation genes (Stucken et al., 2010), whereas in the case of *Chrysosporum* and *Umezakia*, the difference in morphology may be tied to the environmental conditions in which they grow. Either of these causes (genetic determination or morphological variability induced by the environment) could be true for *Nunduva* and *Kyrtuthrix*. *Kyrtuthrix* has become less densely arranged when in culture, like *Nunduva* (Johansen et al., 2021; León‐Tejera et al., 2016), although the U‐shaped loop structure persists for years.

In the modern taxonomy of cyanobacteria, the default option is to trust the molecular signal and downplay morphology, which is often insufficient for recognizing full taxonomic diversity (Bohunická et al., 2024; Pietrasiak et al., 2019, 2021). This case is very difficult because it does not follow current trends. Indeed, the authors of this manuscript were not in agreement as to whether we should collapse *Nunduva* into *Kyrtuthrix* or keep both genera separate. We have opted to keep both genera separate for now because the sample size was small and the evidence was conflicting. We would like to see more strains isolated, and we would especially like to see genomes of the two genera sequenced, to better inform a decision to retain or combine them. Targeted PCR of the ribosomal operons and sequencing of many clones to better understand the number, sequence, and secondary structure of the ITS rRNA regions within species could also help in the revision of species and genera in this group of marine cyanobacteria.

## AUTHOR CONTRIBUTIONS

**Alžběta Vondrášková:** Conceptualization (lead); data curation (lead); formal analysis (supporting); investigation (lead); writing – original draft (lead); writing – review and editing (equal). **Tomáš Hauer:** Data curation (supporting); formal analysis (supporting); investigation (supporting). **Jan Mareš:** Formal analysis (supporting); investigation (equal); writing – review and editing (equal). **Esther Berrendero‐Gomez:** Investigation (supporting). **Jan Zima Jr.:** Investigation (supporting). **Haydee Montoya‐Terreros:** Investigation (equal). **Chelsea D. Villanueva:** Formal analysis (supporting); investigation (supporting); methodology (supporting). **Jeffrey R. Johansen:** Formal analysis (lead); writing – review and editing (lead).

## Supporting information


**Figure S1.** Three gene BI analysis tree used to constrain analysis shown in Figures 4 and S2. Genes used were 16S rRNA, *rbc*L, and *rpo*C1. BI posterior probabilities (* = 1.00) and ML bootstrap values ≥50% are mapped to nodes (* = 100%).

**Figure S2.** Uncollapsed ML phylogeny based on 16S rRNA sequence with outgroup taxa shown, constrained to agree with the topology of the three‐gene tree. This is the uncollapsed source tree for Figure 4. ML bootstrap values ≥50% (* = 100%) and BI posterior probabilities (* = 1.00) and are mapped to nodes.

**Figure S3.** Predicted secondary structures from operon 1, marked with graphical representation of paired nucleotide categorization showing structures recovered from *Kyrtuthrix dalmatica, Nunduva sanagustinensis, Calothrix parasitica, Nunduva* sp. PCC 7116, *Nunduva sanctimaloensis*, and *Nunduva britannica*. Core basal clamp sequence highlighted in gray, 5′–3′ CG pairs highlighted in purple, GC pairs in green, UA pairs in blue, AU pairs in pink, and non‐canonical GU/UG pairs in yellow. Canonical pairings for each structure are summed at the top. Closely related structures, nearest relatives or sister taxa, marked with double ended arrows. Non‐canonical pairings categorized per canonical pairs at the exact loci of closely related structures, are marked with alpha and beta designations. From left to right structures range from most ancestral to most derived.

**Figure S4.** DIV scores and lengths in nucleotides based on the D1–D1′ helices. Note there was little variation in DIV scores, but operons were separable by length.

**Table S1.** Percent similarity of the 16S rRNA gene among *Kyrtuthrix* and *Nunduva* strains. *Nunduva fasciculata* is represented by three different strains, and the similarity to that species is a mean using all three strains. All strains have PS ≥ 97.0%, evidence for collapsing the genera into a single genus. Strains with PS ≥ 99.5% possibly belong to the same species (gray shading), and those with PS ≤ 98.7% are supported as separate species. *Kyrtuthrix* strains are highlighted in green, while *Nunduva* strains are highlighted in yellow. Average intraspecific percent similarity in *N. fasciculata* = 99.1%.

**Table S2.** Percent dissimilarity among ITS rRNA regions in *Kytuthrix* and *Nunduva* strains, arranged in table by operon type. All between strain comparisons have PD ≥ 6.7%, evidence for accepting all strains as different species. Operon 1 sequences are highlighted in blue, while operon 2 sequences are highlighted in dull orange. Variable sequences among operon types within single strains are shown for *Calthrix parasitica* and *N. biania*.

**Table S3.** Alignment of D1–D1′ helix in *Kyrtuthrix* and *Nunduva*, based on secondary structural pairing, showing existence of two different operons. Bases in same color on opposite sides of the terminal loop (center, blue shading) pair. The top six sequences representing one operon additionally pair in the center, breaking the terminal loop into a smaller terminal loop and subterminal loop, whereas the bottom 11 sequences represent the second operon which has a single, larger terminal loop. See Figure 5 in the main text for diagrammatic representation of secondary structure of the D1–D1′ helix.
