# Supplementary figures and images for: Molecular characterization of the type species of Kyrtuthrix (Rivulariaceae, Cyanobacteriota) with comparison to Nunduva: Morphologically different but molecularly cryptic genera
Source: J Phycol. 2025 Jul 28;61(5):1274–87. doi: 10.1111/jpy.70063 (PMC12547636; doi:10.1111/jpy.70063)

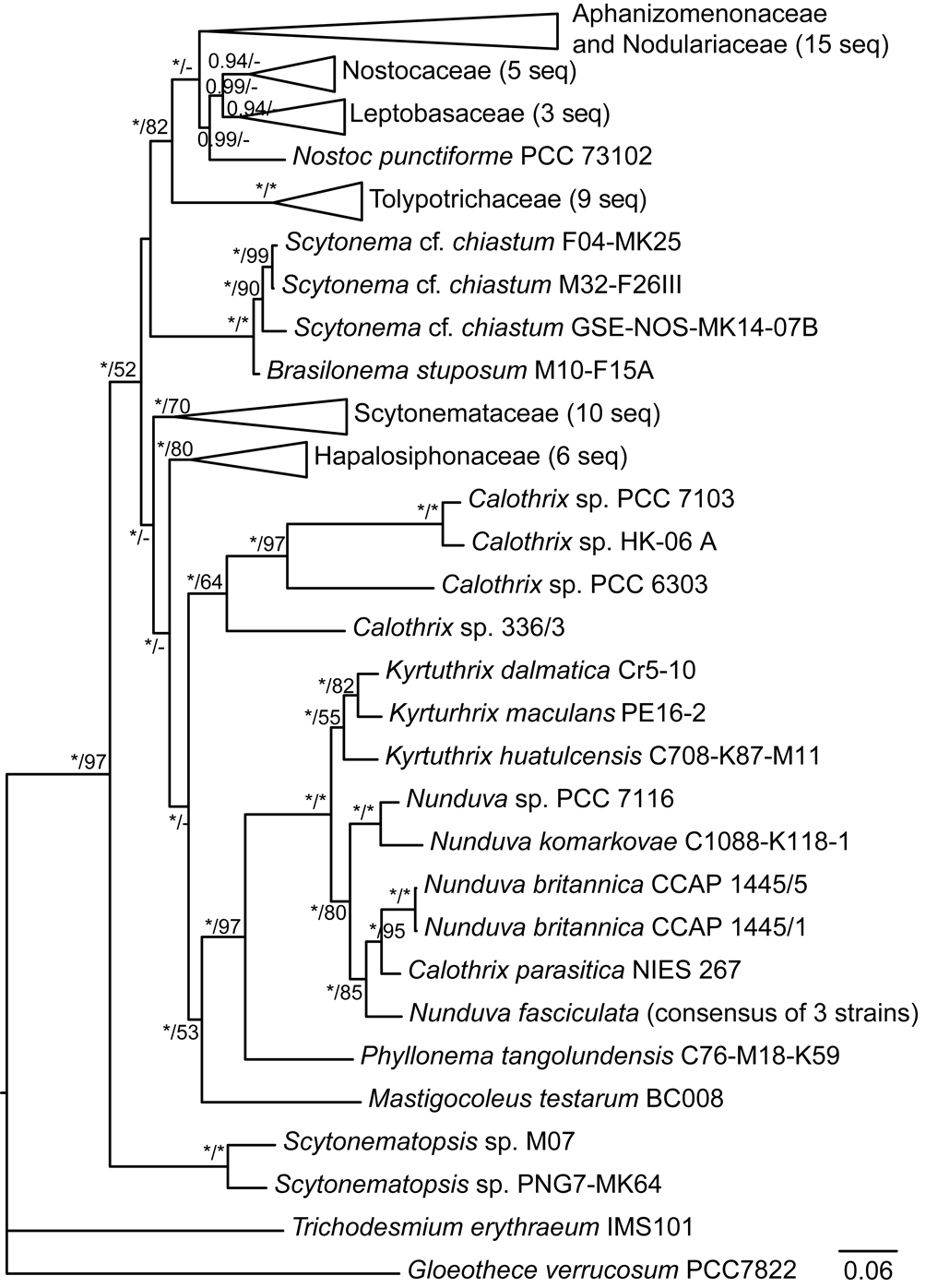

Supplement: Supplementary file 1 — Figure S1. Three gene BI analysis tree used to constrain analysis shown in Figures 4 and S2. Genes used were 16S rRNA, rbcL, and rpoC1. BI posterior probabilities (* = 1.00) and ML bootstrap values ≥50% are mapped to nodes (* = 100%). [file JPY-61-1274-s005.tif]

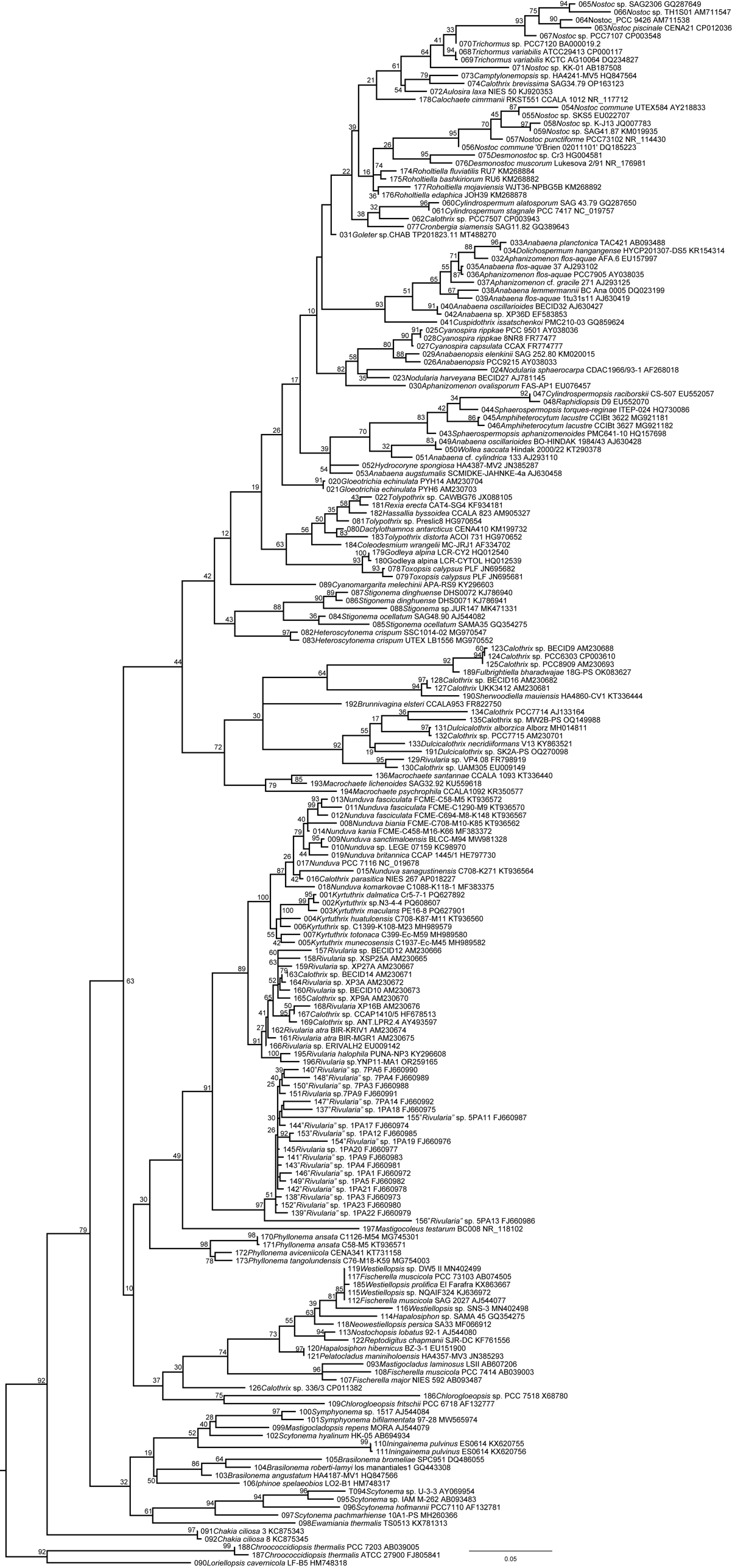

Supplement: Supplementary file 2 — Figure S2. Uncollapsed ML phylogeny based on 16S rRNA sequence with outgroup taxa shown, constrained to agree with the topology of the three‐gene tree. This is the uncollapsed source tree for Figure 4. ML bootstrap values ≥50% (* = 100%) and BI posterior probabilities (* = 1.00) and are mapped to nodes. [file JPY-61-1274-s004.tif]

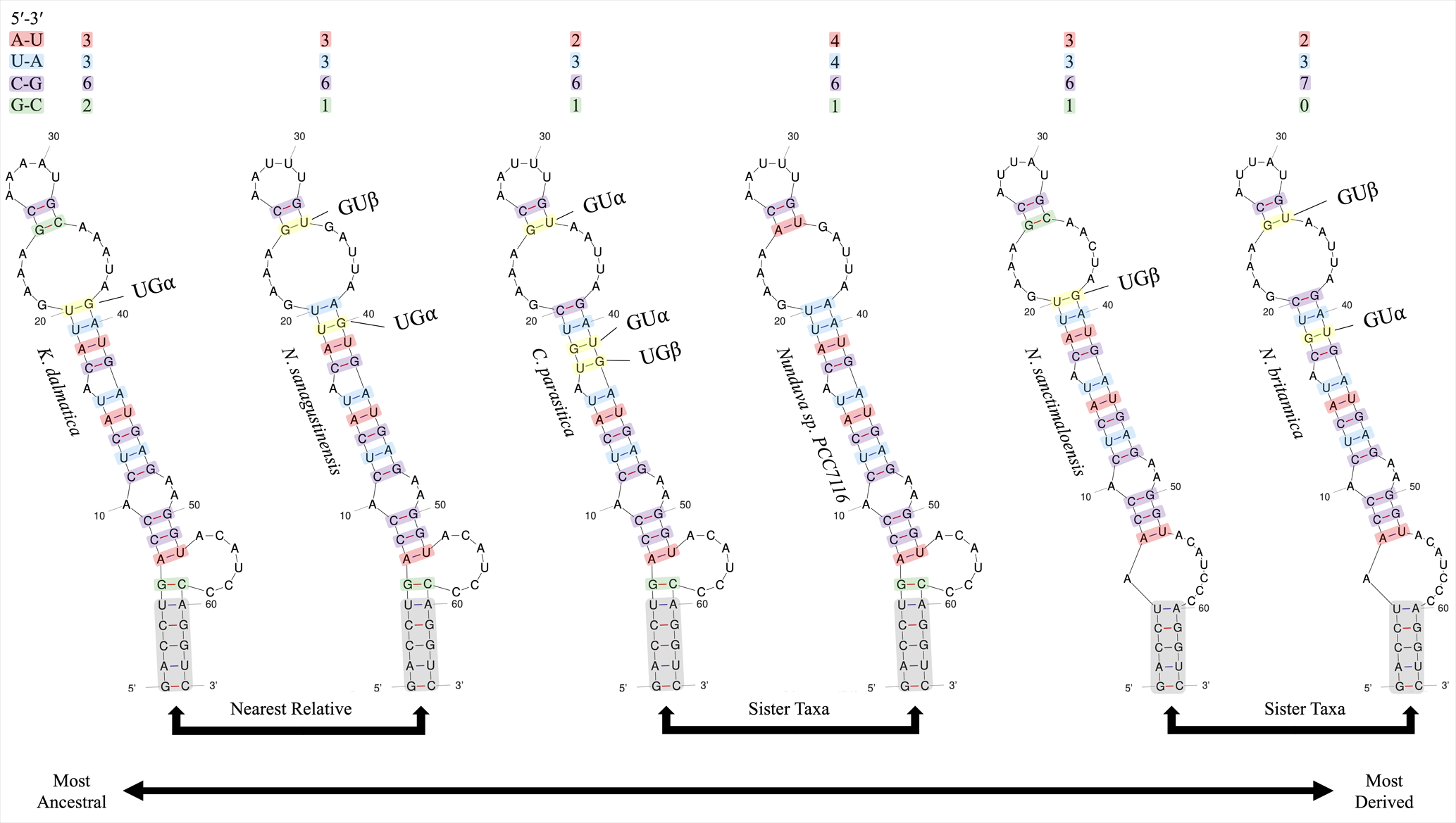

Supplement: Supplementary file 3 — Figure S3. Predicted secondary structures from operon 1, marked with graphical representation of paired nucleotide categorization showing structures recovered from Kyrtuthrix dalmatica, Nunduva sanagustinensis, Calothrix parasitica, Nunduva sp. PCC 7116, Nunduva sanctimaloensis, and Nunduva britannica. Core basal clamp sequence highlighted in gray, 5′–3′ CG pairs highlighted in purple, GC pairs in green, UA pairs in blue, AU pairs in pink, and non‐canonical GU/UG pairs in yellow. Canonical pairings for each structure are summed at the top. Closely related structures, nearest relatives or sister taxa, marked with double ended arrows. Non‐canonical pairings categorized per canonical pairs at the exact loci of closely related structures, are marked with alpha and beta designations. From left to right structures range from most ancestral to most derived. [file JPY-61-1274-s007.tif]

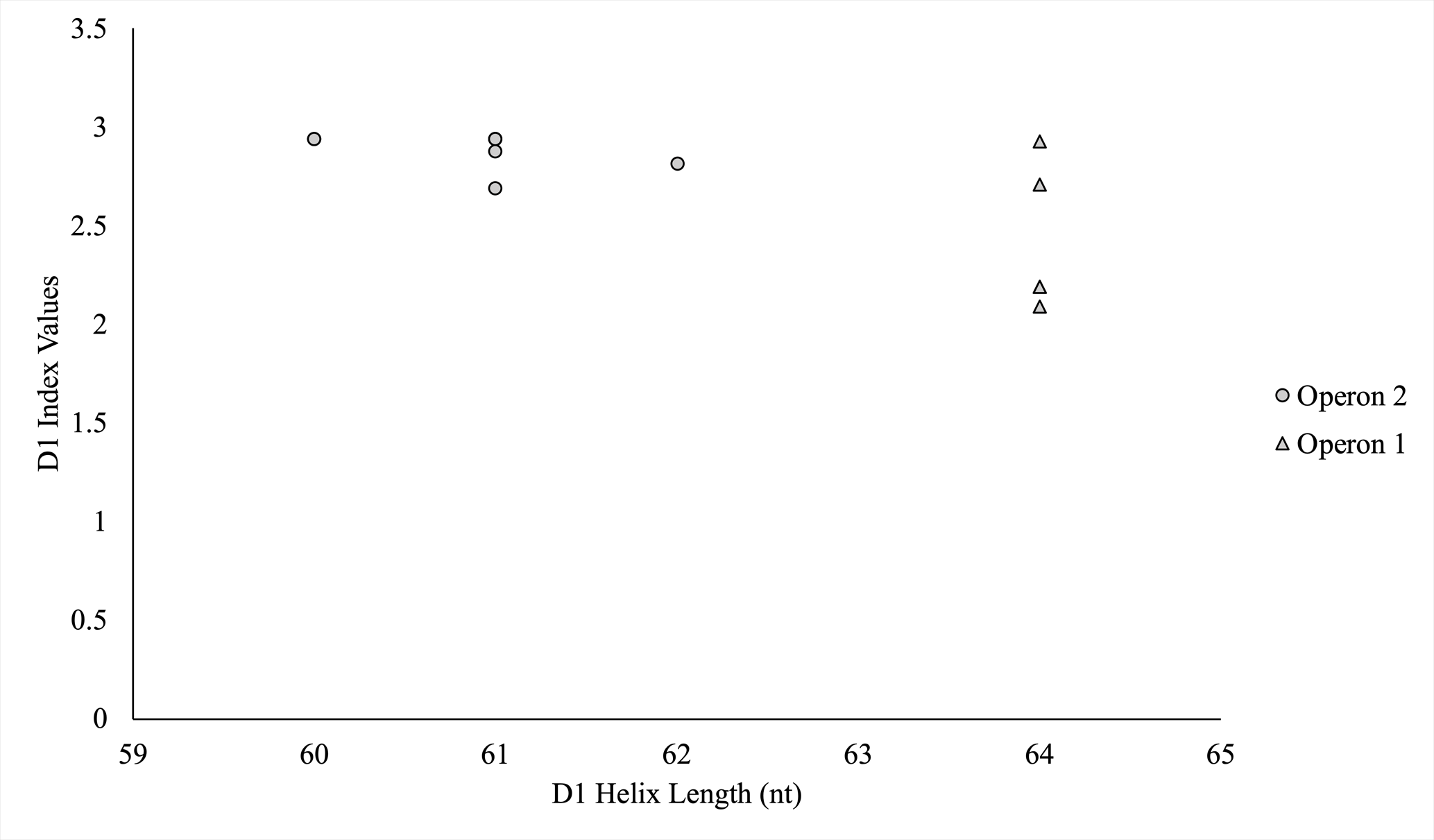

Supplement: Supplementary file 4 — Figure S4. DIV scores and lengths in nucleotides based on the D1–D1′ helices. Note there was little variation in DIV scores, but operons were separable by length. [file JPY-61-1274-s001.tif]
