# Supplementary material for: Molecular characterization of the type species of Kyrtuthrix (Rivulariaceae, Cyanobacteriota) with comparison to Nunduva: Morphologically different but molecularly cryptic genera
Source: J Phycol. 2025 Jul 28;61(5):1274–87. doi: 10.1111/jpy.70063 (PMC12547636; doi:10.1111/jpy.70063)
Supplement: Supplementary file 5 — Table S1. Percent similarity of the 16S rRNA gene among Kyrtuthrix and Nunduva strains. Nunduva fasciculata is represented by three different strains, and the similarity to that species is a mean using all three strains. All strains have PS ≥ 97.0%, evidence for collapsing the genera into a single genus. Strains with PS ≥ 99.5% possibly belong to the same species (gray shading), and those with PS ≤ 98.7% are supported as separate species. Kyrtuthrix strains are highlighted in green, while Nunduva strains are highlighted in yellow. Average intraspecific percent similarity in N. fasciculata = 99.1%. [file JPY-61-1274-s003.docx]

Table S1. Percent similarity of the 16S rRNA gene among *Kyrtuthrix* and *Nunduva* strains. *Nunduva fasciculata* is represented by three different strains, and the similarity to that species is a mean using all three strains. All strains have PS ≥ 97.0%, evidence for collapsing the genera into a single genus. Strains with PS ≥ 99.5% possibly belong to the same species (gray shading), and those with PS≤98.7% are supported as separate species. *Kyrtuthrix* strains are highlighted in green, while *Nunduva* strains are highlighted in yellow. Average intraspecific percent similarity in *N. fasciculata* = 99.1%.

|  | *Kyrtuthrix dalmatica* | *Kyrtuthrix maculans* | *Kyrtuthrix* sp. N3-4-4 | *Kyrtuthrix huatulcensis* | *Kyrtuthrix munecosensis* | *Kyrtuthrix totonaca* | *Kyrtuthrix* sp. C1399 | *Nunduva biania* | *Nunduva sanctimaloensis* | *Nunduva* sp. LEGE 07159 | *Calothrix parasitica* NIES 267 | *Nunduva* sp. PCC7116 | *Nunduva kania* | *Nunduva fasciculata* (mean) | *Nunduva sanagustinensis* | *Nunduva komarkovae* |
| --- | --- | --- | --- | --- | --- | --- | --- | --- | --- | --- | --- | --- | --- | --- | --- | --- |
| *Kyrtuthrix dalmatica* |  |  |  |  |  |  |  |  |  |  |  |  |  |  |  |  |
| *Kyrtuthrix maculans* | 99.5 |  |  |  |  |  |  |  |  |  |  |  |  |  |  |  |
| *Kyrtuthrix* sp. N3-4-4 | 99.7 | 99.4 |  |  |  |  |  |  |  |  |  |  |  |  |  |  |
| *Kyrtuthrix huatulcensis* | 98.2 | 98.2 | 98.1 |  |  |  |  |  |  |  |  |  |  |  |  |  |
| *Kyrtuthrix munecosensis* | 98.5 | 98.5 | 98.4 | 98.8 |  |  |  |  |  |  |  |  |  |  |  |  |
| *Kyrtuthrix totonaca* | 98.1 | 98.1 | 98.0 | 98.9 | 98.9 |  |  |  |  |  |  |  |  |  |  |  |
| *Kyrtuthrix* sp. C1399 | 98.6 | 98.6 | 98.6 | 99.0 | 99.1 | 98.8 |  |  |  |  |  |  |  |  |  |  |
| *Nunduva biania* | 98.2 | 97.8 | 98.1 | 97.2 | 97.3 | 97.0 | 97.6 |  |  |  |  |  |  |  |  |  |
| *Nunduva sanctimaloensis* | 98.4 | 98.2 | 98.3 | 97.7 | 97.9 | 97.7 | 98.0 | 98.9 |  |  |  |  |  |  |  |  |
| *Nunduva* sp. LEGE 07159 | 98.6 | 98.4 | 98.5 | 97.7 | 97.9 | 97.5 | 98.2 | 98.5 | 99.6 |  |  |  |  |  |  |  |
| *Calothrix parasitica* NIES 267 | 99.1 | 98.9 | 99.0 | 97.9 | 98.1 | 98.0 | 98.2 | 98.6 | 99.3 | 98.8 |  |  |  |  |  |  |
| *Nunduva* sp. PCC7116 | 99.3 | 98.9 | 99.2 | 98.3 | 98.4 | 98.2 | 98.6 | 98.5 | 99.1 | 98.9 | 99.6 |  |  |  |  |  |
| *Nunduva kania* | 99.0 | 98.7 | 98.9 | 98.1 | 98.0 | 97.7 | 98.1 | 98.9 | 99.1 | 99.0 | 99.1 | 99.4 |  |  |  |  |
| *Nunduva fasciculata* (mean) | 98.2 | 97.8 | 98.1 | 97.3 | 97.6 | 97.2 | 97.6 | 98.2 | 98.4 | 98.5 | 98.2 | 98.6 | 98.5 |  |  |  |
| *Nunduva sanagustinensis* | 97.7 | 97.3 | 97.7 | 97.4 | 97.1 | 97.9 | 97.4 | 97.2 | 97.6 | 97.4 | 98.1 | 98.0 | 97.6 | 97.2 |  |  |
| *Nunduva komarkovae* | 98.2 | 98.2 | 98.3 | 97.7 | 97.6 | 98.2 | 97.9 | 97.7 | 98.3 | 97.9 | 98.5 | 98.7 | 98.6 | 97.9 | 97.8 |  |
| *Nunduva britannica* | 99.2 | 99.0 | 99.1 | 97.6 | 97.8 | 97.4 | 98.4 | 98.2 | 98.6 | 99.0 | 98.6 | 98.9 | 99.1 | 98.4 | 97.2 | 97.9 |
