# Supplementary material for: Molecular characterization of the type species of Kyrtuthrix (Rivulariaceae, Cyanobacteriota) with comparison to Nunduva: Morphologically different but molecularly cryptic genera
Source: J Phycol. 2025 Jul 28;61(5):1274–87. doi: 10.1111/jpy.70063 (PMC12547636; doi:10.1111/jpy.70063)
Supplement: Supplementary file 6 — Table S2. Percent dissimilarity among ITS rRNA regions in Kytuthrix and Nunduva strains, arranged in table by operon type. All between strain comparisons have PD ≥ 6.7%, evidence for accepting all strains as different species. Operon 1 sequences are highlighted in blue, while operon 2 sequences are highlighted in dull orange. Variable sequences among operon types within single strains are shown for Calthrix parasitica and N. biania. [file JPY-61-1274-s002.docx]

Table S2b. Percent dissimilarity among ITS rRNA regions in *Kytuthrix* and *Nunduva* strains, arranged in table by operon type. All between strain comparisons have PD ≥ 6.7%, evidence for accepting all strains as different species. Operon 1 sequences are highlighted in blue, while operon 2 sequences are highlighted in dull orange. Variable sequences among operon types within single strains are shown for *C. parasitica* and *N. biania*.

|  | *K. dalmatica* operon 1 | *K. maculans* operon 1 | *N. sanctimaloensis* operon 1 | *N. britannica* operon 1 | *Nunduva* sp. PCC7116 operon 1 | *N. sanagustinensis* operon 1 | *C. parasitica* operon 1a | *C. parasitica* operon 1b | *K. hualtescensis* operon 2 | *K. totonaca* operon 2 | *K. munecosensis* operon 2 | *Kyrtuthrix* sp. operon 2 | *N. komarkovae* operon 2 | *N. kania* operon 2 | *N.. fasciculata* operon 2 | *N. biana* KT936561 operon 2a |
| --- | --- | --- | --- | --- | --- | --- | --- | --- | --- | --- | --- | --- | --- | --- | --- | --- |
| *K. dalmatica* operon 1 |  |  |  |  |  |  |  |  |  |  |  |  |  |  |  |  |
| *K. maculans* operon 1 | 10.9 |  |  |  |  |  |  |  |  |  |  |  |  |  |  |  |
| *N. sanctimaloensis* operon 1 | 8.5 | 9.1 |  |  |  |  |  |  |  |  |  |  |  |  |  |  |
| *N. britannica* operon 1 | 9.9 | 12.4 | 11.0 |  |  |  |  |  |  |  |  |  |  |  |  |  |
| *Nunduva* sp. PCC7116 operon 1 | 12.2 | 15.9 | 12.8 | 16.8 |  |  |  |  |  |  |  |  |  |  |  |  |
| *N. sanagustinensis* operon 1 | 9.9 | 10.2 | 9.8 | 14.2 | 12.7 |  |  |  |  |  |  |  |  |  |  |  |
| *C. parasitica* operon 1a | 14.2 | 17.3 | 12.1 | 15.2 | 11.9 | 13.2 |  |  |  |  |  |  |  |  |  |  |
| *C. parasitica* operon 1b | 14.8 | 17.6 | 11.8 | 15.5 | 9.4 | 14.7 | 4.5 |  |  |  |  |  |  |  |  |  |
| *K. hualtescensis* operon 2 | 20.9 | 18.4 | 19.5 | 21.4 | 24.0 | 21.8 | 22.8 | 25.7 |  |  |  |  |  |  |  |  |
| *K. totonaca* operon 2 | 23.3 | 20.4 | 18.7 | 23.9 | 22.5 | 24.5 | 20.7 | 22.3 | 13.3 |  |  |  |  |  |  |  |
| *K. munecosensis* operon 2 | 22.5 | 19.9 | 20.3 | 23.5 | 24.5 | 24.7 | 21.7 | 22.3 | 12.0 | 12.2 |  |  |  |  |  |  |
| *Kyrtuthrix* sp. operon 2 | 22.0 | 19.0 | 18.9 | 22.5 | 21.6 | 23.4 | 20.3 | 21.0 | 7.7 | 12.2 | 6.7 |  |  |  |  |  |
| *N. komarkovae* operon 2 | 18.7 | 16.0 | 16.3 | 20.2 | 18.2 | 15.8 | 18.6 | 20.9 | 14.6 | 21.0 | 20.5 | 18.6 |  |  |  |  |
| *N. kania* operon 2 | 18.1 | 14.8 | 16.0 | 19.7 | 16.7 | 17.8 | 18.2 | 20.3 | 15.8 | 19.7 | 18.8 | 17.2 | 12.0 |  |  |  |
| *N.. fasciculata* operon 2 | 17.3 | 14.8 | 16.7 | 18.7 | 19.7 | 20.0 | 19.6 | 20.2 | 14.8 | 18.7 | 19.4 | 17.7 | 14.3 | 12.6 |  |  |
| *N. biana* KT936561 operon 2a | 12.3 | 9.8 | 11.2 | 15.3 | 16.1 | 13.5 | 18.4 | 19.4 | 17.0 | 18.2 | 19.1 | 17.4 | 14.3 | 15.3 | 14.9 |  |
| *N. biana* KT936562 operon 2b | 12.7 | 10.8 | 11.3 | 16.3 | 17.4 | 14.0 | 17.4 | 18.5 | 17.0 | 16.5 | 17.7 | 16.6 | 14.7 | 15.7 | 15.4 | 3.1 |
