# Supplementary material for: Molecular characterization of the type species of Kyrtuthrix (Rivulariaceae, Cyanobacteriota) with comparison to Nunduva: Morphologically different but molecularly cryptic genera
Source: J Phycol. 2025 Jul 28;61(5):1274–87. doi: 10.1111/jpy.70063 (PMC12547636; doi:10.1111/jpy.70063)
Supplement: Supplementary file 7 — Table S3. Alignment of D1–D1′ helix in Kyrtuthrix and Nunduva, based on secondary structural pairing, showing existence of two different operons. Bases in same color on opposite sides of the terminal loop (center, blue shading) pair. The top six sequences representing one operon additionally pair in the center, breaking the terminal loop into a smaller terminal loop and subterminal loop, whereas the bottom 11 sequences represent the second operon which has a single, larger terminal loop. See Figure 5 in the main text for diagrammatic representation of secondary structure of the D1–D1′ helix. [file JPY-61-1274-s006.docx]

Table S3. Alignment of D1-D1’ helix in *Kyrtuthrix* and *Nunduva*, based on secondary structural pairing, showing existence of two different operons. Bases in same color on opposite sides of the terminal loop (center, blue shading) pair. The top six sequences representing one operon additionally pair in the center, breaking the terminal loop into a smaller terminal loop and subterminal loop, whereas the bottom eleven sequences represent the second operon which has a single, larger terminal loop. See Figure 5 in the main text for diagrammatic representation of secondary structure of the D1-D1’ helix.

_____________________________________________________________________________________________________________________________________

*K. dalmatica* GACCTGACC-ACTCATACATTGAAAGCAAAATGCAAATAGATGATGAG---AAGGTACATCCC-AGGTC

*N. britanica* GACCTAACC-ACTCATACGTCGAAAGCATTATGTAATTAGATGATGAG---AAGGTACATCCC-AGGTC

*C. parasitica* GACCTGACC-ACTCATATGTCGAAAGCAATTTGTAATTAGATGATGAG---AAGGTACATCCC-AGGTC

*N. sanctimaloensis*  GACCTAACC-ACTCATACATTGAAAGCATTATGCAACTAGATGATGAG---AAGGTACATCCC-AGGTC

*Nunduva* sp. PCC7116 GACCTGACC-ACTCATACATTGAAAACAATTTGTGATTAAATGATGAG---AAGGTACATCCC-AGGTC

*N. sanagustinensis* GACCTGACC-ACTCATACATTGAAAGCAATTTGTGATTAAGTGATGAG---AAGGTACATCCC-AGGTC

*K. maculans* ??????????????????????????????????????????????????????????ATCCC-AGGTC

*K. hualtescensis* GACCTTACCCATTCATACACCGAGC---ATTG--ATATAGGTGATGATTGT--GGT-CATCCCAAGGTC

*K. totonaca* GACCTTACCCACTCATACACCGAGA---TATT--ATATAGGTGATGATTGT--GGT-CATCCCAAGGTC

*K. munecosensis* GACCT-ACCCACTCATACACCGAGC---ATTG--ATATAGGTGATGATTGT--GGT-CATCCCAAGGTC

*Kyrtuthrix* sp GACCTTACCCATTCATACACCGAGC---ATTG--ATATAGGTGATGATTGT--GGT-CATCCCAAGGTC

*N. komarkovae* GACCTTACCCATTCATACATCTTCC---GATT-GTTATAGATAATGATTGT--GGT-CATCCCAAGGTC

*N. kania* GACCTTACCCATTCATACATCGAGA----ATT-GATATAGATGATGATTGT--GGT-CATCCCAAGGTC

*N. fasciculata* GACCTTACCCATTCATACACCGAGA---TAT---ATATAGGTGATGATTGT--GGT-CATCCCAAGGTC

*N. fasciculata* GACCTTACCCATTCATGCACCGAGA---TAT---ATATAGGTGATGATTGT--GGT-CATCCCAAGGTC

*N. fasciculata* GACCTTACCCATTCATACACCGAGG---TAT---ATATAGGTGATGATTGT--GGT-CATCCCAAGGTC

*N. biania* GACCTTACCCATTCATACACCGAAA---AATA--ATATAGGTGATGATTGT--GGT-CATCCCAAGGTC

*N. biania* GACCTTACCCATTCATACACCGAAA---AATA--ATATAGGTGATGATTGT--GGT-CATCCCAAGGTC

_______________________________________________________________________________________________________________
